# Intravascular Large B-Cell Lymphoma Diagnosed by Open Brain Biopsy and Achievement of Remission After Early Initiation of Chemotherapy: Case Report

**DOI:** 10.7759/cureus.21971

**Published:** 2022-02-07

**Authors:** Eisuke Tsukamoto, Takafumi Tanei, Takenori Kato, Toshinori Hasegawa

**Affiliations:** 1 Neurosurgery, Komaki City Hospital, Komaki, JPN

**Keywords:** mri, microbleeds, brain, biopsy, intravascular large b-cell lymphoma

## Abstract

A 60-year-old man presented with progressive disturbance of consciousness. His father had died of malignant lymphoma, while his mother and sister died of acute leukemia. Magnetic resonance imaging (MRI) revealed multiple high-intensity lesions in the bilateral cerebral hemispheres on diffusion-weighted images. Serum soluble interleukin 2 receptor was 5,640 U/mL. Screenings of blood antibodies known to rise in autoimmune diseases were all normal. Cerebrospinal fluid examinations demonstrated slight elevation of protein and glucose, while the oligoclonal band and myelin basic protein were not elevated. Biopsies of bone marrow and random skin did not show any malignant features. His consciousness gradually deteriorated over a week, with lesions in his right frontal, left temporal, and bilateral parietal lobes shown to be growing. Therefore, open brain biopsy was performed, and one block of the right frontal lesion was harvested. Histological examination revealed atypical large cells only in the capillaries. Although immunohistochemical examinations showed positive staining for CD20, they were negative for CD3. Histopathological diagnosis was intravascular large B-cell lymphoma. After undergoing six cycles of intravenous chemotherapy with rituximab, cyclophosphamide, doxorubicin, and prednisone, his consciousness and neurological symptoms improved, and he appeared to achieve remission. Two years later, there have been no apparent recurrences, and the brain lesions have disappeared.

## Introduction

Intravascular large B-cell lymphoma (IVLBCL) is a rare subtype of extranodal diffuse large B-cell lymphoma that is characterized by selective growth of lymphoma cells within the lumina of small blood vessels [1-3]. IVLBCL diagnosis requires performing select tissue biopsies, such as that of bone marrow, skin, lung, spleen, or brain. However, the diagnosis is frequently difficult due to the initial non-specific symptoms and absence of non-invasive diagnostic markers. In addition, IVLBCL progresses rapidly, with a delay of diagnosis often resulting in fatal outcomes. Here, we describe a case of IVLBCL diagnosed by open brain biopsy and subsequent achievement of remission due to early initiation of chemotherapy.

## Case presentation

**Figure 1:**
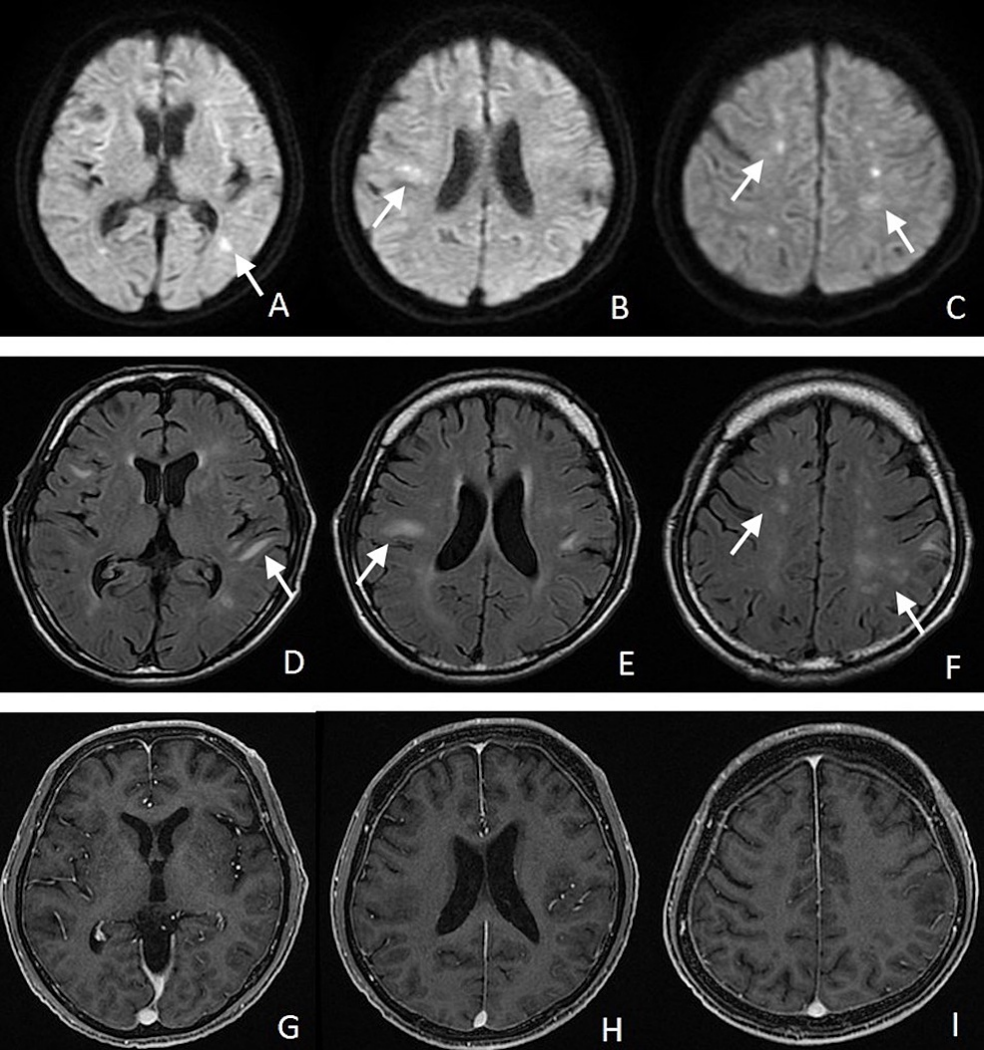
Magnetic resonance images on arrival Magnetic resonance images show the multiple lesions in the bilateral cerebral hemispheres as hyperintensity on the diffusion-weighted (A-C) and fluid-attenuated inversion recovery images (D-F). The lesions did not show any enhancements on contrast-enhanced T1-weighted images (G-I).

A 60-year-old man had a history of ischemic stroke and was undergoing dialysis because of unexplained chronic kidney disease. There was a history of cancer in his family, with his father dying of malignant lymphoma and his mother and sister dying of acute leukemia. He was transported to our hospital due to subacute progressive disturbance of consciousness. Magnetic resonance imaging (MRI) revealed multiple small high-intensity lesions in the bilateral cerebral hemispheres on diffusion-weighted and fluid-attenuated inversion recovery images (Figures 1A-1F). These lesions did not show any enhancements on contrast-enhanced T1-weighted images (Figures 1G-1I). Initially, as these findings were suspected to be due to ischemic strokes, he was started on antiplatelet therapy. Serum soluble interleukin 2 receptor was 5,640 U/mL. Screenings of blood antibodies about autoimmune diseases were all normal. Although cerebrospinal fluid examinations demonstrated a slight elevation of protein, the oligoclonal band and myelin basic protein were not elevated.

**Figure 2:**
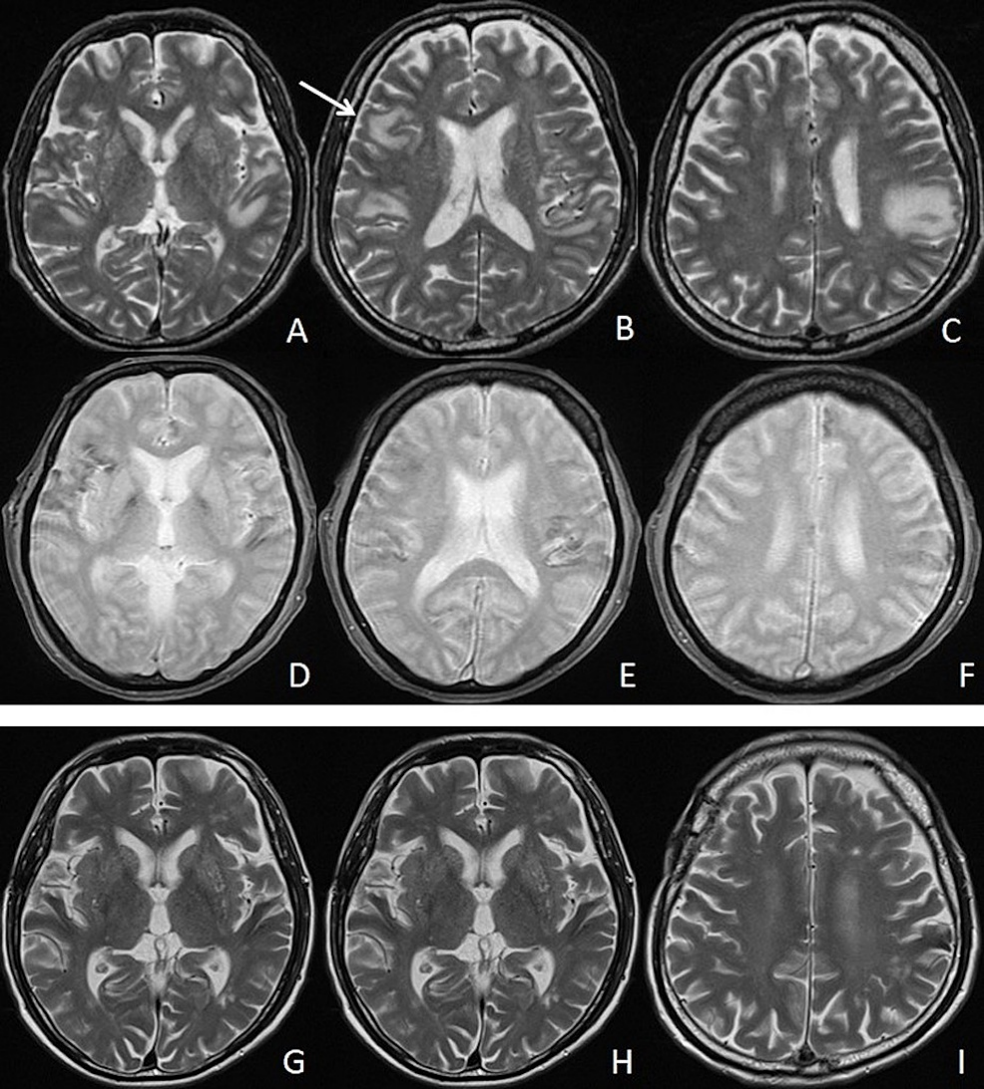
Subsequent magnetic resonance images Subsequent magnetic resonance images obtained one week later showed an increase in the high-intensity lesions in the right frontal, left temporal, and bilateral parietal lobes on T2-weighted images (A-C). The specimen was harvested from the right frontal lesion (B, arrow). Hemosiderosis was detected around these lesions as low intensity on the T2*-weighted image (D-F). Two years later, high-intensity lesions had almost disappeared on the T2-weighted images (G-I).

A bone marrow biopsy was performed, but the first pathological results did not show any malignant features. In addition, a random skin biopsy did not find any atypical lymphocytes. In spite of the antiplatelet therapy, his consciousness gradually deteriorated over a week. A subsequent MRI showed increases in the high-intensity lesions in the right frontal, left temporal, and bilateral parietal lobes on T2-weighted images (Figures 2A-2C). Furthermore, we detected multiple low intensities at the surface of the cortex around these lesions on the T2*-weighted images (Figures 2D-2F). Therefore, an open brain biopsy of the right frontal lobe was planned in an attempt to determine an early diagnosis. After confirming the location of the right frontal lesion by the navigation system, a small craniotomy was performed (Figure 2B).

**Figure 3:**
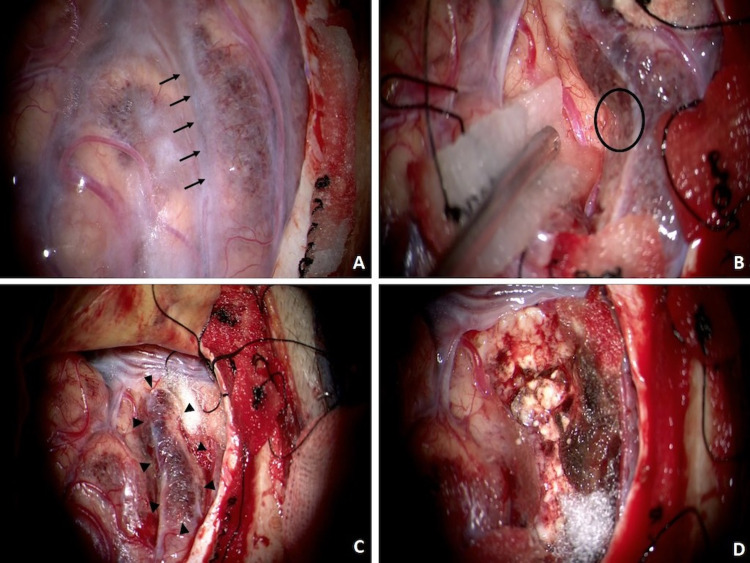
Intraoperative photographs Intraoperative photographs show that the surface of the lesion was covered by hemosiderin (A). The sulcus between the lesion and normal cortex was separated (A, arrows), with a small tissue sample that was fragile and easily bled then harvested (B, black circle). An adequate size of specimen was harvested as one block of the lesion (C, arrowheads, D).

The surface of the lesion was covered by hemosiderin (Figure 3A). The sulcus between the lesion and normal cortex was separated, followed by the harvesting of a small tissue sample, which was fragile and easily bled (Figure 3B). However, the intraoperative consultation of the specimen revealed no abnormalities. Therefore, larger specimen size was harvested as one block of the lesion (Figures 3C, 3D).

**Figure 4:**
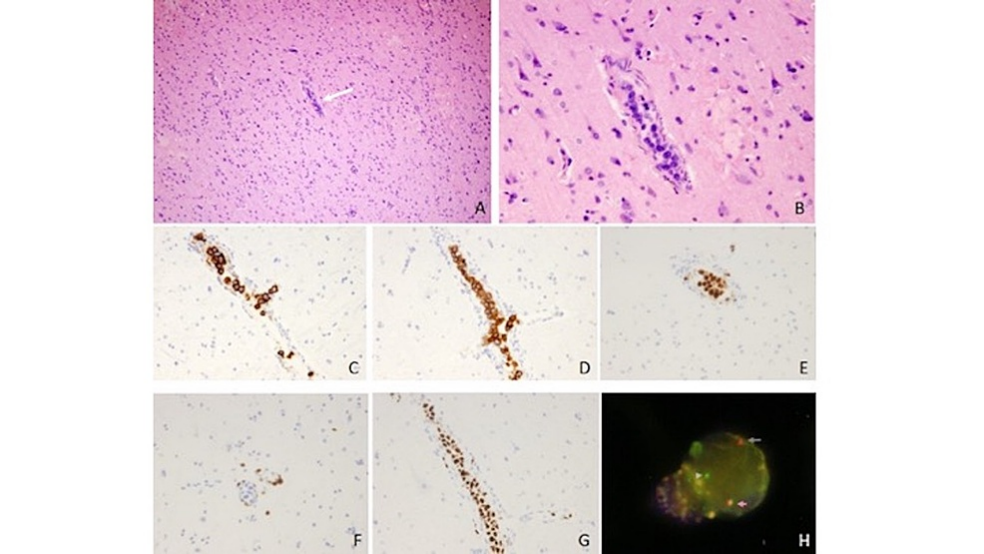
Surgical pathological specimen Surgical specimen stained with hematoxylin and eosin showed atypical large cells only in the capillaries (A: x100, B: x400). Immunohistochemical examinations showed that these atypical cells exhibited positive staining for CD20 (C), CD79a (D), and MUM1 (E), but were negative for CD3 (F). Mib-1 index was high (G). Translocation of B-cell lymphoma 6 was detected using a FISH analysis. Red and green signals were independent, which revealed the signal of transduction (H). FISH, fluorescence in situ hybridization; MUM1, multiple myeloma 1

Histological examination revealed atypical large cells only in the capillaries without any of these cells found around the parenchymal tissue (Figures 4A, 4B). Immunohistochemical examinations demonstrated that while the atypical cells stained positive for CD20, CD79a, and multiple myeloma 1 (MUM1), they were negative for CD3 (Figures 4C-4F). Mib-1 index was high (Figure 4G). The translocation of B-cell lymphoma 6 (BCL6) was detected using a FISH (fluorescence in situ hybridization) analysis (Figure 4H). The histopathological diagnosis was IVLBCL. Final results of the bone marrow biopsy, which was completed after the biopsy, showed the existence of a few atypical large B cells. These findings supported the diagnosis of IVLBCL.

After diagnosis, the patient was treated with six cycles of intravenous chemotherapy with rituximab, cyclophosphamide, doxorubicin, and prednisone (R-CHOP). His consciousness and neurological symptoms improved, and he appeared to achieve remission. Two years later, there have been no apparent recurrences, and the high-intensity lesions of the MRI have almost completely disappeared (Figure 2G-2I).

## Discussion

IVLBCL is now divided into three types (classical, cutaneous, and hemophagocytic syndrome-associated) according to the clinical features [4]. The classic type presents with many non-specific signs and symptoms, such as fever of unknown origin and involvement of the central nervous system and skin. It has been reported that 24-42% of IVLBCL patients develop neurological symptoms during the course of the diseases [5,6]. The cutaneous type with better prognosis is limited to skin lesions without other systemic involvement. The worst prognosis is seen for the hemophagocytic syndrome-associated type, which presents with cytokine release syndrome including splenomegaly and/or hepatomegaly [4]. The pathological diagnosis of IVLBCL is made by demonstrating the excessive proliferation of lymphoma cells within the lumina of small vessels and capillaries, without the involvement of adjacent parenchymal tissue [1,2]. The lymphoma cells are mainly large cells with prominent nucleoli and mitotic figures [1,3]. Immunohistological analysis has shown that the lymphoma cells are positive for CD20, CD45, CD79a, and MUM1, while they are negative for CD3 [7-10]. Translocation of the *BCL6* gene in IVLBCL has also been reported [11].

Although biopsies were performed for each of the affected organs, they were initially only performed for the bone marrow or skin. According to the report, 67% patients were diagnosed with IVLBCL by bone marrow biopsy. [12]. Random skin biopsy is less invasive than other biopsies and is therefore considered as a standard diagnostic procedure [13]. The random skin biopsy diagnosis rate has been reported to be 69% [13]. The use of a skin biopsy is reasonable, especially for the cutaneous type of IVLBCL. The classical type of IVLBCL has been reported to primarily present with neurological symptoms such as brain lesions without any skin lesions. Thus, it is possible that bone marrow and skin biopsies in these types of cases could potentially not determine a correct pathological diagnosis. In other previously reported cases, brain biopsies were required as other biopsy methods were not able to definitively determine a diagnosis, even though the brain lesions exhibited growth or the development of an enhanced mass [7-10]. All of these previous brain biopsy cases were performed using a craniotomy and not via a stereotactic needle [7-10].

Matsue et al. presented MR images that showed abnormal findings in 32 (86.4%) of 37 IVLBCL patients [13]. There are five abnormal patterns that are observed in these patients: (1) infarct-like lesions, (2) non-specific white matter lesions, (3) meningeal enhancement, (4) mass-like lesions, and (5) hyperintense lesions in the pons on a T2-weighted image [14]. In our case, the MR images initially showed infarct-like lesions in the bilateral cerebral hemispheres. Subsequently, these lesions began to grow and the MRI findings changed to non-specific white matter and mass-like lesions. The intraoperative view showed that there was a hemosiderosis surface of the cortex around the lesions, which was consistent with the findings of the T2*-weighted images. The hemosiderosis indicated microbleeds from the lesions. There have been several previous studies that have reported finding multiple microbleeds around the brain lesions on T2*-weighted or susceptibility-weighted images [8,10,15]. The biopsy from the bleeding site showed that there were multiple ruptures of small vessels, in which large clusters of cells existed [16]. These findings for IVLBCL on MRI are new features and could potentially be helpful in making a differential diagnosis.

As IVLBCL diagnosis is difficult due to the non-specific symptoms, IVLBCL is often diagnosed by autopsy. However, if it were possible to diagnose IVLBCL, chemotherapy for IVLBCL could potentially be effective [2,6]. Rajyaguru et al. reported that the five-year survival rate for treated patients was approximately 60%, with this rate better than the 46.4% five-year survival rate reported in the Surveillance, Epidemiology, and End Results database [17]. Matsue et al. examined IVLBCL patients in whom an in vivo diagnosis was possible and reported that 60% of the patients survived for more than five years when administered combination chemotherapy [13]. In addition, they also reported that of the 40 patients who responded favorably to R-CHOP, these patients fully recovered without any focal neurological deficit except for three out of four neurolymphomatosis patients [12]. Standard R-CHOP chemoimmunotherapy without the use of high-dose methotrexate and/or cytarabine is an adequate and active therapy with favorable toxicity for patients suffering from CNS manifestations of IVLBCL [3].

## Conclusions

In our case, several factors indicated neoplastic brain lesions, such as a family history of cancer, atypical cerebral infarction findings on MRI, lesions that appeared to be growing, deterioration of the patient’s consciousness over a short period, and elevation of the serum soluble interleukin-2 receptor. Although bone marrow and random skin biopsies were promptly performed, the first biopsy results did not show any malignant features. At that time, IVLBCL was given as one of differential diagnosis. It could be pathologically undiagnosed when only using a small specimen obtained via a stereotactic needle biopsy, as IVLBCL diagnosis requires proof of atypical cells in small blood vessels. Therefore, in our case, we planned to perform an open brain biopsy, which could select harvest both small and large size specimens. Even though our initial small specimen revealed no abnormalities, the large size of the specimen subsequently made a diagnosis. As has been reported in other studies, an early pathological diagnosis along with an early initiation of chemotherapy can make it possible for patients to achieve remission for IVLBCL. Our current results show that open brain biopsy can be a useful choice for IVLBCL diagnosis when there are brain lesions present and other biopsy methods cannot definitively determine a correct diagnosis.
